# Non-invasive diagnosis of esophageal cancer by a simplified circulating cell-free DNA methylation assay targeting OTOP2 and KCNA3: a double-blinded, multicenter, prospective study

**DOI:** 10.1186/s13045-024-01565-2

**Published:** 2024-06-18

**Authors:** Yan Bian, Ye Gao, Han Lin, Chang Sun, Wei Wang, Siyu Sun, Xiuling Li, Zhijie Feng, Jianlin Ren, Hezhong Chen, Chaojing Lu, Jinfang Xu, Jun Zhou, Kangkang Wan, Lei Xin, Zhaoshen Li, Luowei Wang

**Affiliations:** 1 Department of Gastroenterology, Changhai Hospital, Naval Medical University, Shanghai, China; 2 Changhai Clinical Research Unit, Changhai Hospital, Naval Medical University, Shanghai, China; 3 National Key Laboratory of Immunity and Inflammation, Naval Medical University, Shanghai, China; 4 Department of Gastroenterology, Shengjing Hospital, China Medical University, Shenyang, Liaoning China; 5 Department of Gastroenterology, Henan Provincial People’s Hospital, Zhengzhou, Henan China; 6 Department of Gastroenterology, The Second Hospital of Hebei Medical University, Hebei Medical University, Shijiazhuang, Hebei China; 7 Department of Gastroenterology, Zhongshan Hospital, Xiamen University, Xiamen, Fujian China; 8 Department of Thoracic Surgery, Changhai Hospital, Naval Medical University, Shanghai, China; 9 Department of Health Statistics, Naval Medical University, Shanghai, China; 10 Wuhan Ammunition Life-Tech Company, Ltd., Wuhan, Hubei China

**Keywords:** Liquid biopsy, Cell-free DNA, Methylation, Esophageal cancer, Noninvasive detection

## Abstract

**Background:**

Esophageal cancer (EC) is a highly lethal disease lacking early detection approaches. We previously identified that OTOP2 and KCNA3 were specifically hypermethylated in circulating cell-free DNA from patients with EC. We then developed a blood-based methylation assay targeting OTOP2 and KCNA3 (named “IEsohunter”) for esophageal cancer noninvasive detection. This double-blinded, multicenter, prospective study aimed to comprehensively evaluate its clinical diagnostic performance.

**Methods:**

Participants with EC, high-grade intraepithelial neoplasia (HGIN), other malignancies, benign gastrointestinal lesions, or no abnormalities were prospectively enrolled from 5 tertiary referral centers across China. Peripheral blood samples were collected, followed by plasma cell-free DNA methylation analysis using the IEsohunter test based on multiplex quantitative polymerase chain reaction adopting an algorithm-free interpretation strategy. The primary outcome was the diagnostic accuracy of IEsohunter test for EC.

**Results:**

We prospectively enrolled 1116 participants, including 334 patients with EC, 71 with HGIN, and 711 controls. The areas under the receiver operating characteristic curves of the IEsohunter test for detecting EC and HGIN were 0.903 (95% CI 0.880–0.927) and 0.727 (95% CI 0.653–0.801), respectively. IEsohunter test showed sensitivities of 78.5% (95% CI 69.1–85.6), 87.3% (95% CI 79.4–92.4), 92.5% (95% CI 85.9–96.2), and 96.9% (95% CI 84.3–99.8) for stage I-IV EC, respectively, with an overall sensitivity of 87.4% (95% CI 83.4–90.6) and specificity of 93.3% (95% CI 91.2–94.9) for EC detection. The IEsohunter test status turned negative (100.0%, 47/47) after surgical resection of EC.

**Conclusions:**

The IEsohunter test showed high diagnostic accuracy for EC detection, indicating that it could potentially serve as a tool for noninvasive early detection and surveillance of EC.

**Supplementary Information:**

The online version contains supplementary material available at 10.1186/s13045-024-01565-2.

To the editor:

Esophageal cancer (EC) has a poor prognosis, with a less than 30% five-year overall survival rate [1, 2]. This is largely due to almost 80% of patients with EC being diagnosed at an advanced stage [3, 4]. Upper gastrointestinal endoscopy with targeted biopsy is not suitable for mass screening of the general population due to its invasiveness, resource limitations, and relatively low prevalence of target lesions. Novel blood-based biomarkers, such as cell-free DNA (cfDNA), cell-free RNA, metabolites, and proteins, have demonstrated potential in the early detection of EC [5–11]. However, these classifiers typically use algorithms to combine various biomarkers such as logistic regression, random forest regression, and lasso regression, challenges persist in model validation, results interpretation, and clinical applications.

In our previous study, we identified OTOP2 and KCNA3 by whole-genome bisulfite sequencing as promising DNA-methylation biomarkers for EC diagnosis [12]. Given the purpose of simplifying the diagnostic model and further validation, it is necessary to provide sufficient evidence to develop a ready-to-use diagnostic test with acceptable accuracy for the screening and early detection for patients with EC.

Between September 7, 2022, and October 19, 2023, 1173 participants underwent screening, with the final 1116 being enrolled (Fig. 1A). The study included three participant groups: high-grade intraepithelial neoplasia (HGIN, n = 71), EC (n = 334), and control (n = 711). The control group can be subdivided into healthy control (HC, n = 65), gastrointestinal benign lesion (BL, n = 483), and other malignancies (n = 163), where other malignancies further consist of digestive system malignancies (DSM, n = 82) and non-digestive system malignancies (Non-DSM, n = 81). Clinicopathological characteristics of participants are summarized in Fig. 1B. Patients enrollment, clinical and laboratory procedures are described in Supplementary Material 1.

**Fig. 1:**
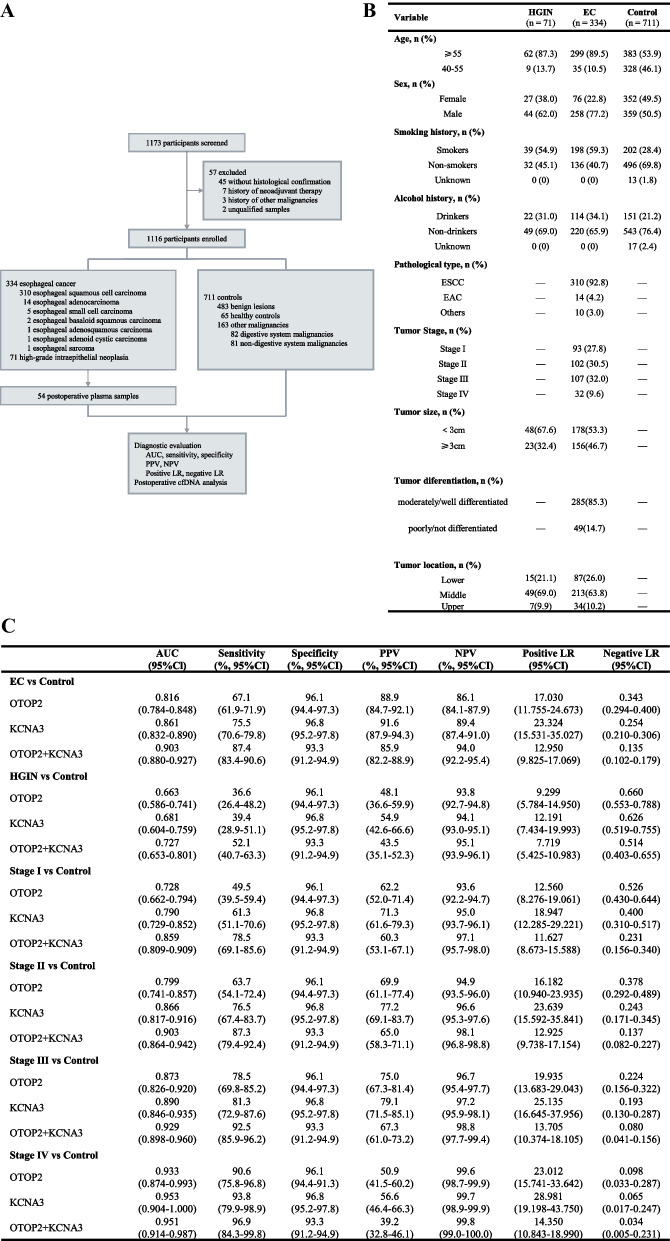
**A** Study profile. **B** The characteristics of participants. **C** Results for measurement of plasma methylated OTOP2, KCNA3, or either, in the diagnosis of EC. AUC = The area under the receiver operating characteristic curve. PPV = positive predictive value. NPV = negative predictive value. LR = likelihood ratio. cfDNA = cell-free DNA. EC = esophageal cancer, HGIN = high-grade intraepithelial neoplasia.

Plasma methylated OTOP2 and KCNA3 cycle threshold (Ct) values were significantly lower in patients with esophageal squamous cell carcinoma (ESCC) than in all controls (*P* < 0.0001; Fig. 2A, B; TableS1, S2). Similarly, both esophageal adenocarcinoma (EAC) and other histopathologic types of EC also differed significantly from all controls. There were no significant differences in values among the three EC groups including ESCC, EAC and other EC (Fig. 2A, B). It should be noted that the number of EAC and other EC cases enrolled was low due to the incidence, and more studies are needed to further investigate the methylation levels and diagnostic accuracy of KCNA3 and OTOP2 for EAC and other EC. Besides, HGIN was also significantly different from all control groups (*P* < 0.0001; Fig. 2A, B).

**Fig. 2:**
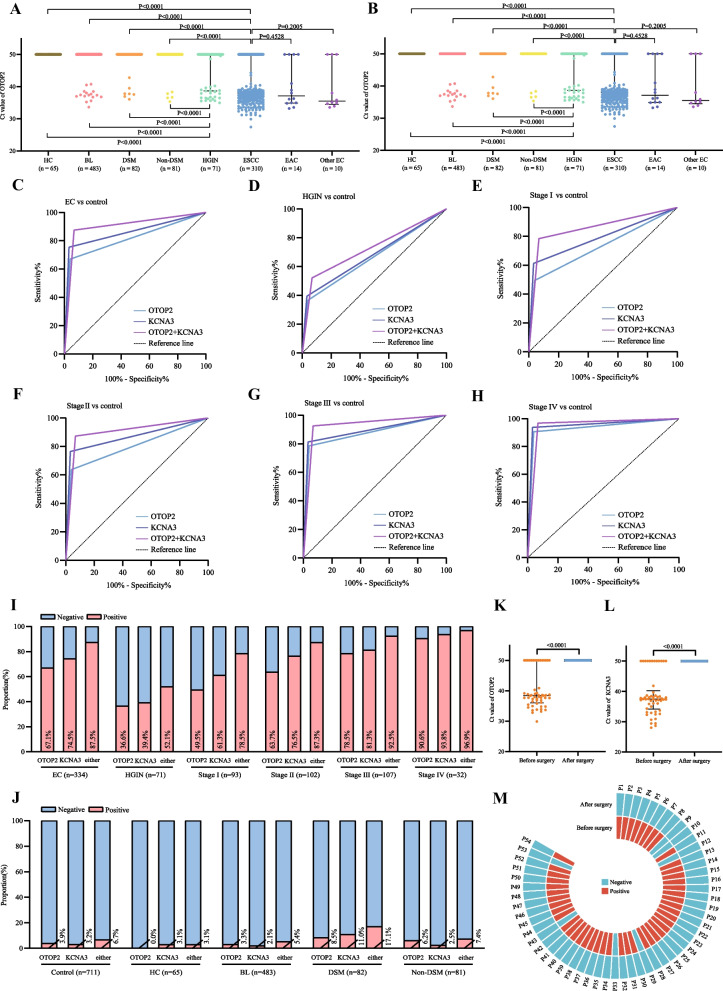
**A** Ct values of plasma methylated OTOP2 for the participants. **B** Ct values of plasma methylated KCNA3 for the participants. **C** ROC for OTOP2, KCNA3, or either for all patients with EC versus all controls. **D** ROC for OTOP2, KCNA3, or either for patients with HGIN versus all controls. **E** ROC for OTOP2, KCNA3, or either for patients with stage I EC versus all controls. **F** ROC for OTOP2, KCNA3, or either for patients with stage II EC versus all controls. **G** ROC for OTOP2, KCNA3, or either for patients with stage III EC versus all controls. **H** ROC for OTOP2, KCNA3, or either for patients with stage IV EC versus all controls. **I** The proportion of positive results for OTOP2, KCNA3, or either, in all patients with EC and patients with HGIN, stage I, stage II, stage III, and stage IV EC. **J** The proportion of positive results for OTOP2, KCNA3, or either, in all controls, HC, and patients with BL, DSM, and non-DSM. Either of KCNA3 and OTOP2 positive was defined as positive, and both negative was defined as negative. **K** Ct value of plasma methylated OTOP2 in patients with EC before and one day after surgery. **L** Ct value of plasma methylated KCNA3 in patients with EC before and one day after surgery. **M** Annular heatmap of the result of combining OTOP2 and KCNA3 in paired plasma samples from before and one day after surgery from the same patients with EC. Black horizontal lines are median and error bars are interquartile range. Ct = cycle threshold. ROC = the receiver operating characteristics curve. EC = esophageal cancer. ESCC = esophageal squamous cell carcinoma. EAC = esophageal adenocarcinoma. HGIN = high-grade intraepithelial neoplasia. HC = healthy control. BL = benign lesion. DSM = digestive system malignancy. P = patient

The receiver operating characteristic curve (ROC) analysis showed the IEsohunter test with an algorithm-free interpretation strategy could differentiate EC from all controls (Figs. 1C, 2C–H). In particular, the IEsohunter test exhibited exceptional diagnostic performance for stage I EC (Figs. 1C, 2E, I, Figure S1). Furthermore, the IEsohunter test exhibited similar efficacy in detecting various pathological types of EC and had a relatively low tested positivity proportion in malignancies that were non-EC (Fig. 2J, Table S3). Besides, this panel could accurately differentiate HGIN from controls (Figs. 1C, 2D, I). Predictive values and likelihood ratios for the IEsohunter test in the diagnosis of EC and HGIN were shown in Fig. 1C. The results revealed that using BL, DSM, and Non-DSM as controls led to a slightly reduction in the area under the receiver operating characteristic curve (AUC) compared to using HC as the control (Figure S2, Fig. 2J).

ROC analysis of 5 centers in the diagnosis of EC was shown in Figure S3. The AUC, tested positivity proportions of EC and the controls in different centers were similar. Besides, predictive values and likelihood ratios for different centers were shown in Table S4. In additional stratified analyses, there were no significant differences in the sensitivity of detecting EC using the IEsohunter test when stratified by age, sex, smoking history, alcohol history, tumor differentiation, and tumor location (Table S5).

Besides, 108 plasma samples were collected from 54 patients with EC before and after surgical resection on one day. The Ct values all exceeded the detection limit after surgical resection (Fig. 2K, L). Conclusively, 47 patients with EC who tested positive before surgery all became negative after surgery (Fig. 2M).

In conclusion, we demonstrated the effectiveness of cfDNA diagnosis for patients with EC by using a simplified cfDNA methylation assay. To our knowledge, this is the first large-scale, multicenter study to report the clinical diagnostic accuracy of cfDNA methylation assay for EC. Our results indicate that the IEsohunter test could potentially be used to diagnose and surveil EC, especially early-stage disease, and will help to address the lack of reliable blood biomarkers for EC in clinics and provide a reference for subsequent studies.

## Supplementary Information

Additional file 1.

Additional file 2.

Additional file 3.

## Data Availability

The datasets analyzed in the current study are available upon reasonable request from the corresponding author.
