# Correction: Non-invasive diagnosis of esophageal cancer by a simplified circulating cell-free DNA methylation assay targeting OTOP_2_ and KCNA_3_: a double-blinded, multicenter, prospective study

**DOI:** 10.1186/s13045-024-01653-3

**Published:** 2024-12-21

**Authors:** Yan Bian, Ye Gao, Han Lin, Chang Sun, Wei Wang, Siyu Sun, Xiuling Li, Zhijie Feng, Jianlin Ren, Hezhong Chen, Chaojing Lu, Jinfang Xu, Jun Zhou, Kangkang Wan, Lei Xin, Zhaoshen Li, Luowei Wang

**Affiliations:** 1 Department of Gastroenterology, Changhai Hospital, Naval Medical University, Shanghai, China; 2 Changhai Clinical Research Unit, Changhai Hospital, Naval Medical University, Shanghai, China; 3 National Key Laboratory of Immunity and Inflammation, Naval Medical University, Shanghai, China; 4 Department of Gastroenterology, Shengjing Hospital, China Medical University, Shenyang, Liaoning China; 5 Department of Gastroenterology, Henan Provincial People’s Hospital, Zhengzhou, Henan China; 6 Department of Gastroenterology, The Second Hospital of Hebei Medical University, Hebei Medical University, Shijiazhuang, Hebei China; 7 Department of Gastroenterology, Zhongshan Hospital, Xiamen University, Xiamen, Fujian China; 8 Department of Thoracic Surgery, Changhai Hospital, Naval Medical University, Shanghai, China; 9 Department of Health Statistics, Naval Medical University, Shanghai, China; 10 Wuhan Ammunition Life-Tech Company, Ltd., Wuhan, Hubei China

**Correction : Journal of Hematology & Oncology (2024) 17:47** 10.1186/s13045-024-01565-2

The original article erroneously presents a duplicate of Figure 2A over Figure 2B. The corrected Figure 2 with correct Figure 2B can be viewed ahead in this Correction article.

**Fig. 2:**
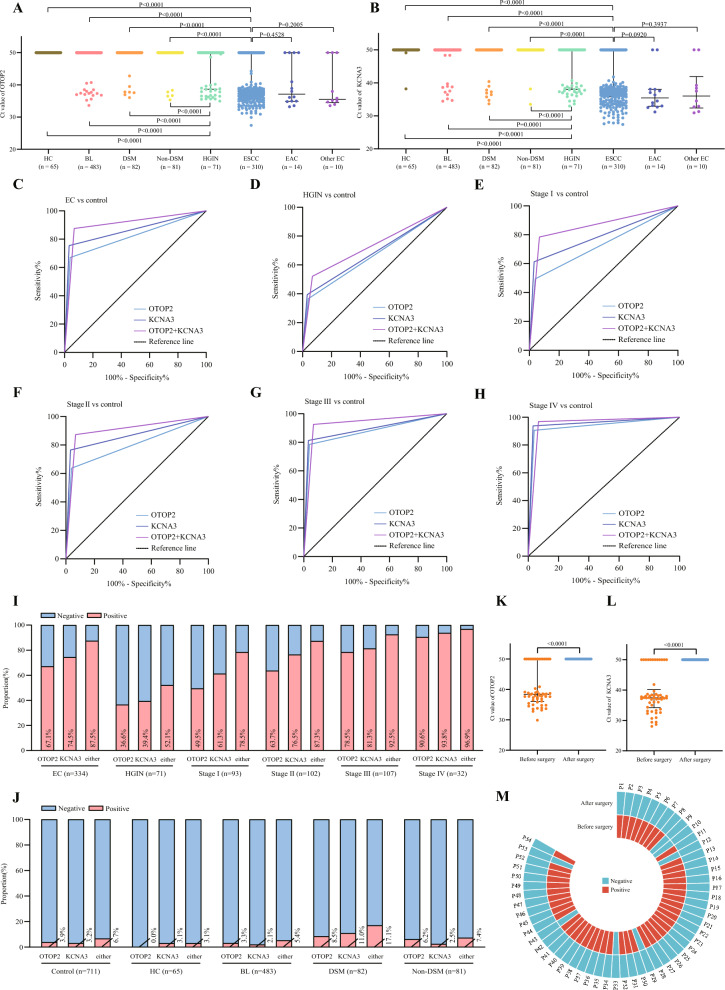
**A** Ct values of plasma methylated OTOP2 for the participants. **B** Ct values of plasma methylated KCNA3 for the participants. **C** ROC for OTOP2, KCNA3, or either for all patients with EC versus all controls. **D** ROC for OTOP2, KCNA3, or either for patients with HGIN versus all controls. **E** ROC for OTOP2, KCNA3, or either for patients with stage I EC versus all controls. **F** ROC for OTOP2, KCNA3, or either for patients with stage II EC versus all controls. **G** ROC for OTOP2, KCNA3, or either for patients with stage III EC versus all controls. **H** ROC for OTOP2, KCNA3, or either for patients with stage IV EC versus all controls. **I** The proportion of positive results for OTOP2, KCNA3, or either, in all patients with EC and patients with HGIN, stage I, stage II, stage III, and stage IV EC. **J** The proportion of positive results for OTOP2, KCNA3, or either, in all controls, HC, and patients with BL, DSM, and non-DSM. Either of KCNA3 and OTOP2 positive was defined as positive, and both negative was defined as negative. **K** Ct value of plasma methylated OTOP2 in patients with EC before and one day after surgery. **L** Ct value of plasma methylated KCNA3 in patients with EC before and one day after surgery. **M** Annular heatmap of the result of combining OTOP2 and KCNA3 in paired plasma samples from before and one day after surgery from the same patients with EC. Black horizontal lines are median and error bars are interquartile range. Ct = cycle threshold. ROC = the receiver operating characteristics curve. EC = esophageal cancer. ESCC = esophageal squamous cell carcinoma. EAC = esophageal adenocarcinoma. HGIN = high-grade intraepithelial neoplasia. HC = healthy control. BL = benign lesion. DSM = digestive system malignancy. P = patient

