# Supplementary material for: Non-invasive diagnosis of esophageal cancer by a simplified circulating cell-free DNA methylation assay targeting OTOP2 and KCNA3: a double-blinded, multicenter, prospective study
Source: J Hematol Oncol. 2024 Jun 18;17:47. doi: 10.1186/s13045-024-01565-2 (PMC11186155; doi:10.1186/s13045-024-01565-2)
Supplement: Supplementary file 1 — Additional file 1. [file 13045_2024_1565_MOESM1_ESM.docx]

**Methods**

**Study population**

We prospectively recruited consecutive healthy controls and patients with esophageal cancer (EC), high-grade intraepithelial neoplasia (HGIN), gastrointestinal benign lesions, and other malignancies to perform diagnostic performance evaluation of the IEsohunter test from 5 centers, from September 2022 to October 2023. The participants were enrolled from Shanghai Changhai Hospital (Shanghai, China), Shengjing Hospital of China Medical University (Liaoning, China), Zhongshan Hospital Affiliated to Xiamen University (Xiamen, China), Henan Provincial People’s Hospital (Henan, China), and the Second Hospital of Hebei Medical University (Hebei, China).

The inclusion criteria were as follows: 1. Individuals who were at a high risk of developing EC and were recommended for endoscopy by clinicians. Specifically, people over the age of 40 who met any of the following conditions were considered to be at high risk for EC, (1) long-term residence in areas with a high incidence of EC; (2) symptoms of upper gastrointestinal discomfort; (3) family history of EC; (4) with precancerous lesions of EC; (5) having other risk factors for EC (smoking, heavy alcohol consumption, squamous cancer history of the head and neck or respiratory tract, etc.). 2. Individuals with a strong suspicion of EC or HGIN as indicated by endoscopy, imaging studies, or pathological biopsy. 3. Patients with benign digestive system diseases who were scheduled for endoscopy or already had endoscopic findings. 4. Patients with untreated malignancies other than EC. Enrolled participants should meet at least one of the inclusion criteria. EC was deﬁned based on upper gastrointestinal endoscopy, CT, or MRI characteristics and was conﬁrmed by histopathology. Tumor stage was deﬁned according to the American Joint Committee on Cancer/Union for International Cancer Control (AJCC/UICC’ 8th edition) tumor staging system. The diagnosis of HGIN was based on histopathology of upper gastrointestinal endoscopy biopsy and endoscopic resection. The control group included healthy controls, gastrointestinal benign lesions, and other malignant diseases. Healthy controls were individuals who underwent digestive endoscopy without any abnormalities and had no history of malignant diseases. The gastrointestinal benign lesions group was benign lesions detected after digestive endoscopy with histopathology of endoscopy biopsy, including esophagitis, gastritis, enteritis, appendicitis, gastric polyps, colorectal polyps, etc. Other malignancies were diagnosed by standard diagnostic procedures and confirmed by histopathology. Other malignancies include digestive system malignancies (gastric cancer, colorectal cancer, liver cancer, pancreatic cancer, cholangiocarcinoma, etc.) and non-digestive system malignancies (thyroid carcinoma, lung cancer, cervical cancer, endometrial cancer, breast cancer, prostate cancer, etc.).

Exclusion criteria were listed below: 1. Patients with malignant tumors received treatment prior to enrollment, including surgical resection, radiotherapy, or chemotherapy. 2. Patients with EC or HGIN combined with other malignancies. 3. Individuals without confirmed diagnosis information. 4. Individuals whose samples were unsuitable for methylation detection or analysis, such as hemolytic samples and invalid samples with ineligible cycle threshold (Ct) values. Subjects meeting any of the above exclusion criteria needed to be excluded.

The study was approved by the institutional ethics review committee at each study center and registered in clinicaltrial.gov (NCT05680077). As required by the committees, informed consent was obtained from participants. This study followed STARD guidelines.

**Study procedures**

For patients with EC, HGIN, or other malignancies, blood samples were collected by trained research nurses one day prior to lesion resection, radiotherapy, chemotherapy or other treatments. Histopathological confirmation for resectable malignancies was obtained through postoperative pathological information, and pathological staging was performed. To evaluate changes in plasma levels of methylated OTOP2 and KCNA3 after surgical resection of EC, blood samples were randomly collected from a subset of patients with EC from 5 centers one day after surgery. For participants with benign gastrointestinal diseases or without abnormalities, blood samples could be taken by the research nurses either before or after they underwent gastrointestinal endoscopy at the endoscopy units. Participants' age, gender, and smoking and drinking history were obtained together with the signed informed consents. After sampling, plasma was isolated immediately from the whole blood samples by centrifugation and stored at -80℃ for the IEsohunter test. The data collection and analysis were carried out independently by 3 researchers. In addition, gastroenterologists and pathologists were unaware of the IEsohunter test result for each participant until unblinding.

**The IEsohunter test procedures**

Assays for cfDNA of methylated OTOP2 and KCNA3 were conducted at each study center. The laboratory researchers were blinded to the clinical and grouping information. The plasma DNA was extracted using the Nucleic Acid Extraction and Purification Kit (Wuhan Ammunition Life-tech Co., Ltd, Wuhan, China) according to the protocol. The purified DNA was then subjected to sodium bisulfite treatment using the Bisulfite Conversion Kit (Wuhan Ammunition Life-tech Co., Ltd., Wuhan, China) following the instructions. The converted DNA was amplified and followed by multiplex quantitative polymerase chain reaction with IEsohunter kit (Wuhan Ammunition Life-tech Co., Ltd., Wuhan, China) using an ABI 7500 Real-Time PCR thermal cycler (Thermo Fisher Scientific, Waltham, USA), LightCycler 480 II instrument (Roche, Basel, Switzerland), or SLAN-96P Real-Time PCR System (Sansure Biotech, Hunan, China). The cycling procedure was set to the following conditions: 5 min at 95°C, 50 cycles of 15 s at 95°C, and 30 s at 60°C. The ACTB was used as an internal control. The primers and probes used in the assays were reported in detail in our previous study^1^. In brief, three primer pairs and three probes were used, targeting OTOP2, KCNA3, and ACTB, respectively (Table S6). During the testing, positive and negative controls should be set as well. The templates of the positive control included 10^3^ copies of plasmid-DNA incorporated with methylated gene regions of KCNA3, OTOP2, and ACTB, respectively. In contrast, the template of the negative control was double distilled water. The Ct values for KCNA3, OTOP2 and ACTB in the positive control well must all be less than or equal to 33 for the IEsohunter assay to be considered valid. In addition, there should be no amplification in the negative control wells. The IEsohunter test employed an algorithm-free interpretation strategy to determine whether the tested subjects were positive or negative. The reference range of ACTB is Ct ≤ 34, and the cut-off Ct values for OTOP2 and KCNA3 were both 48. For a valid sample with a Ct value of ACTB ≤ 34, the sample was considered positive for EC if at least one of the Ct values of KCNA3 and OTOP2 ≤ 48. On the other hand, if a valid sample showed that the Ct values of KCNA3 and OTOP2 were both > 48 or not determined, the sample was considered negative for EC. For ease of presentation and statistical analysis, the undetermined Ct values were assigned a number of 50.

**Study outcomes**

The primary outcome was the diagnostic accuracy of the IEsohunter test for detecting EC, and the secondary outcome was the diagnostic accuracy for HGIN and EC in specific clinical stages. The outcome measures included the area under the receiver operating characteristic curve, sensitivity and specificity, positive and negative predictive values, and positive and negative likelihood ratios.

**Statistical analysis**

Statistical analyses were performed using GraphPad Prism (version 9.0.0), MedCalc (version 20.022), and R software (version 4.2.2). The Mann-Whitney U test was used to test for differences between two independent groups with non-normal distributed numerical data. Receiver operating characteristics curves (ROC) were used to evaluate the corresponding areas under the curves (AUC) with a 95% confidence interval (CI). Sensitivity, specificity, predictive values, and likelihood ratios were calculated using their formulas, respectively. Besides, the sample size was estimated using the formula shown in Table S7.

Methylated OTOP2 and KCNA3 levels in plasma were compared before and after surgical resection in patients with EC using the Wilcoxon matched-pairs signed rank test. P values were calculated using two-sided testing, and statistical significance was determined at a threshold of P < 0.05.

**Reference**

1. Bian Y, Gao Y, Lu C, Tian B, Xin L, Lin H, et al. Genome-wide methylation profiling identified methylated KCNA3 and OTOP2 as promising diagnostic markers for esophageal squamous cell carcinoma. Chin Med J 2023; XX: 1–12.
