# Supplementary material for: Non-invasive diagnosis of esophageal cancer by a simplified circulating cell-free DNA methylation assay targeting OTOP2 and KCNA3: a double-blinded, multicenter, prospective study
Source: J Hematol Oncol. 2024 Jun 18;17:47. doi: 10.1186/s13045-024-01565-2 (PMC11186155; doi:10.1186/s13045-024-01565-2)
Supplement: Supplementary file 2 — Additional file 2. [file 13045_2024_1565_MOESM2_ESM.docx]

# Supplement Figure


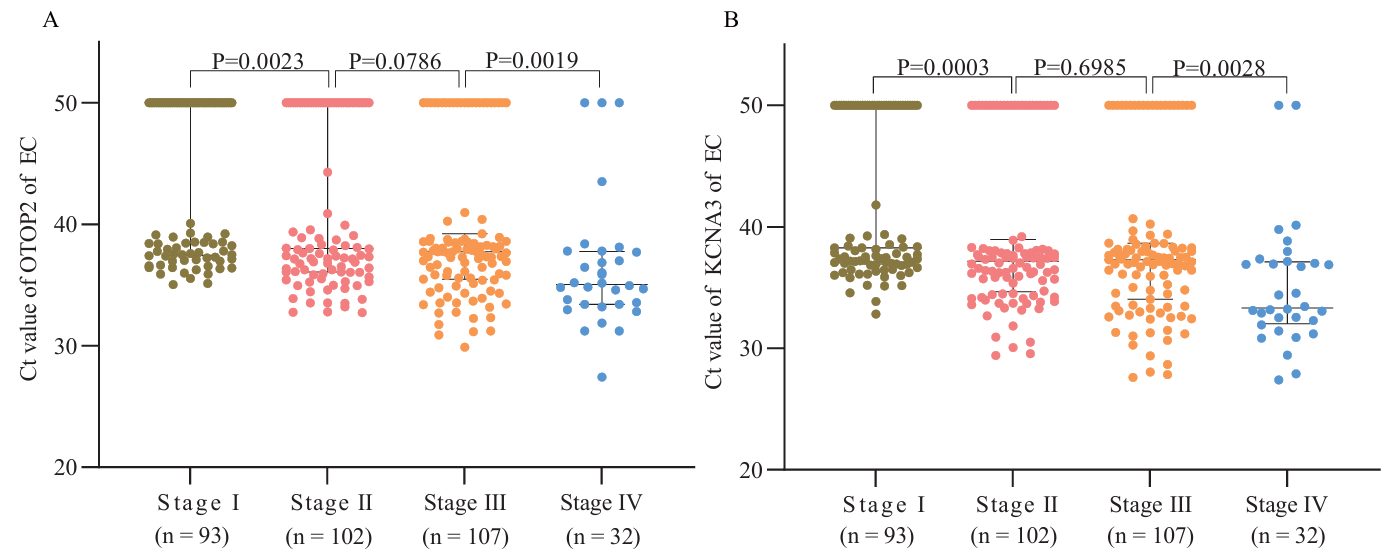


**Figure S1: Ct values of methylated OTOP2 and KCNA3 in plasma in the Stage Ⅰ-Ⅳ EC.**

(A) Methylated OTOP2 for 4 stages of EC. (D) Methylated KCNA3 for 4 stages of EC. Black horizontal lines are median and error bars are interquartile range. Besides, 50 was used as the upper limit for Ct values of OTOP2 and KCNA3.

Ct = cycle threshold. EC=esophageal cancer.


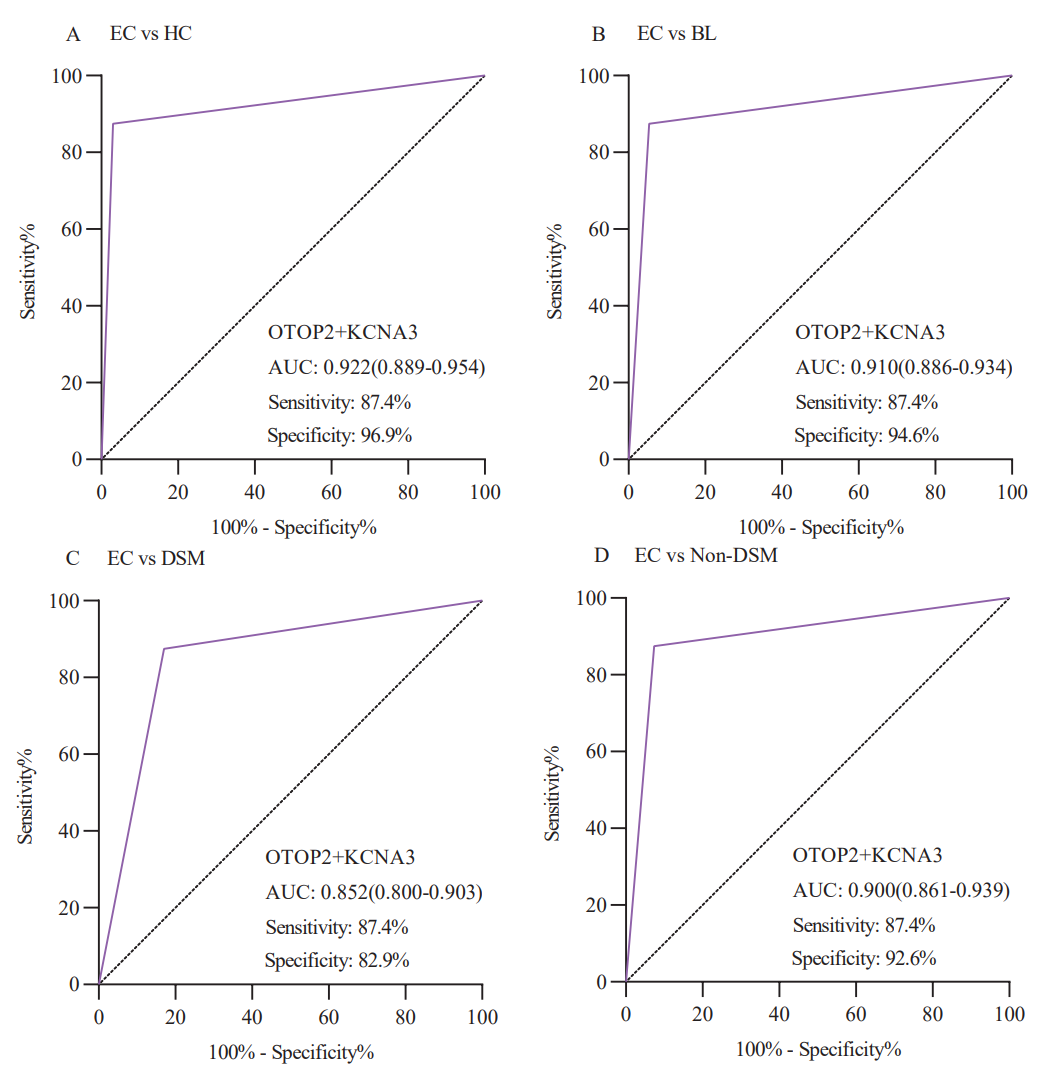


**Figure S2: Diagnostic outcomes for IEsohunter test in the diagnosis of EC versus different controls.**

(A) ROC for IEsohunter test for patients with EC versus HC. (B) ROC for IEsohunter test for patients with EC versus BL. (C) ROC for IEsohunter test for patients with EC versus DSM. (D) ROC for IEsohunter test for patients with EC versus Non-DSM.

ROC = the receiver operating characteristics curve. EC = esophageal cancer. HC = healthy control. BL = benign lesion. DSM = digestive system malignancy.


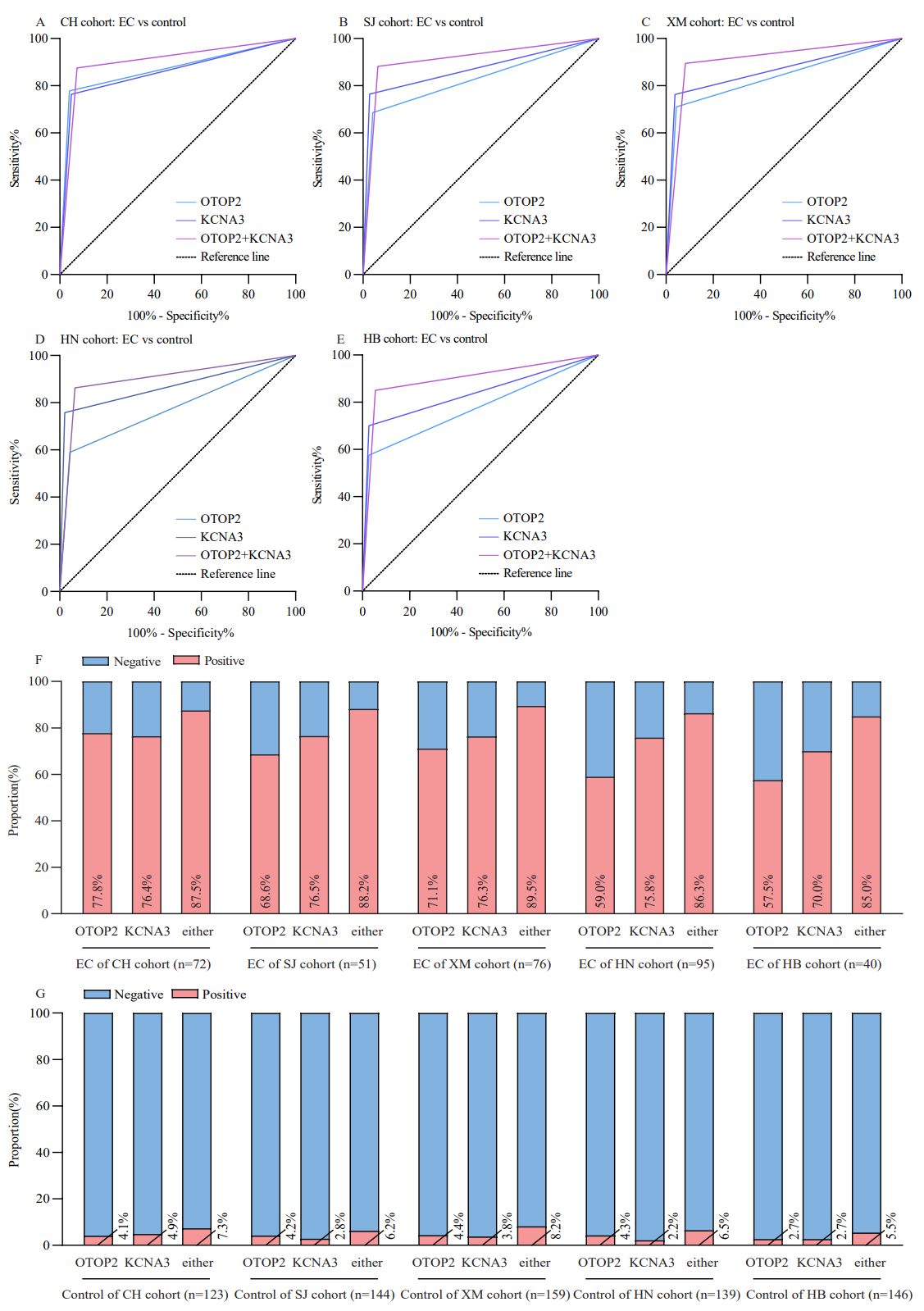


**Figure S3: Diagnostic outcomes for plasma methylated OTOP2 and KCNA3 in the diagnosis of EC in 5 centers.**

(A) ROC for OTOP2, KCNA3, or either for patients with EC versus controls in CH cohort. (B) ROC for OTOP2, KCNA3, or either for patients with EC versus controls in SJ cohort. (C) ROC for OTOP2, KCNA3, or either for patients with EC versus controls in XM cohort. (D) ROC for OTOP2, KCNA3, or either for patients with EC versus controls in HN cohort. (E) ROC for OTOP2, KCNA3, or either for patients with EC versus controls in HB cohort. (F) The proportion of positive results for OTOP2, KCNA3, or either, in patients with EC in CH, SJ, XM, HN, and HB cohorts. (G) The proportion of positive results for OTOP2, KCNA3, or either, in controls in CH, SJ, XM, HN, and HB cohorts. Either of KCNA3 and OTOP2 positive was defined as positive, and both negative was defined as negative.

ROC = the receiver operating characteristics curve. EC = esophageal cancer. CH = Shanghai Changhai Hospital. SJ = Shengjing Hospital of China Medical University. XM = Zhongshan Hospital Affiliated to Xiamen University. HN = Henan Provincial People’s Hospital. HB = The Second Hospital of Hebei Medical University.
